# Effect of Different Combinations of Phosphorus and Nitrogen Fertilization on Arbuscular Mycorrhizal Fungi and Aphids in Wheat

**DOI:** 10.3390/insects11060365

**Published:** 2020-06-11

**Authors:** Chao Wang, Baoliang Tian, Zhenzhen Yu, Jianqing Ding

**Affiliations:** 1 School of Life Sciences, Henan University, Jin Ming Avenue, Kaifeng 475004, Henan, China; wangchao2018henu@163.com (C.W.); tbl007@126.com (Z.Y.); 2 State Key Laboratory of Crop Stress Adaptation and Improvement, Jin Ming Avenue, Kaifeng 475004, Henan, China

**Keywords:** AMF, aphids, fertilization combination, metabolites, plant growth

## Abstract

While chemical fertilizers can be used to increase crop yield, the abuse of fertilizers aggravates environmental pollution and soil degradation. Understanding the effects of chemical fertilizers on the interaction between arbuscular mycorrhizal fungi (AMF) and pest insects is of great benefit to crop and environmental protection, because AMF can enhance the nutrition absorption and insect resistance of crops. This study tested the effect of different levels of phosphorus, nitrogen, and their interactions on AMF, secondary metabolites, *Sitobion avenae* in garden, as well as the wheat traits in field. The results showed that AMF colonization on roots in the P0N1 treatment (0 g P/pot, 1.3083 g N/pot in the garden, and 0 g P/plot, 299.84 g N/plot) was the highest in both the garden and the field. The abundance of aphid was reduced in the P0N1 treatment, and there were negative relationships between aphids and AMF and phenolics, but a positive relationship between AMF and phenolics. Our results indicated that a change in the ratio of phosphorus to nitrogen affects the relationship among AMF, aphid abundance, and metabolites. The results also suggested an approach to save chemical fertilizers that could improve crop health and protect the agroecosystem against pollution at the same time.

## 1. Introduction

Wheat (*Triticum aestivum* L.) is one of most important crops in the world, contributing substantially to global food and nutritional security because of its protein content [1]. The fact that chemical fertilizers can increase crop yields is well known, but the abuse of fertilizers, such as excessive nitrogen (N) or phosphorus (P) fertilization, is not only harmful to yield [2,3] but has also led to groundwater pollution [4], algal bloom [5], and soil acidification [6,7], even resulting in pest outbreak, such as wheat aphids [8]. Therefore, it is important to plant development for suitable soil nutrition. Additionally, there is a negative relationship between soil nutrition (e.g., P) and arbuscular mycorrhizal fungi (AMF) [9,10], which have a strong effect on improving nutrient absorption (e.g., N) and pest resistance [11,12,13,14]. Therefore, it is especially important to determine an appropriate rate of P to N fertilization for the balance between AMF and plant development, which would provide a guide for both suppressing pest insect populations and maintaining crop yield and quality.

Arbuscular mycorrhizal fungi are a widespread mutualist on the terrestrial plants, including the gramineous plant [15,16]. Accumulating research on the relationships between AMF and plants has indicated that AMF can enhance the growth of plants in many ways [12,17]. For example, AMF can facilitate the plant root absorption of soil nutrients (P, N, and microelements), thereby increasing plant growth [17]. Moreover, AMF may enhance the resistance of plants to pest by changing the primary or secondary plant metabolites [18], which have strong antifeedant activity [19] and toxicity [20,21]. For example, a study on the leaf metabolome of willow (*Salix purpurea* L., Salicales: Salicaceae) showed that AMF cause up-regulation of the biosynthetic pathways of isoflavonoids, phenylpropanoids, and chlorophyll synthesis [22]. Other studies indicate that AMF increase the concentration of phenolics or flavonoids in the roots and shoots of plants, and reduce the abundance of legume pod borers (*Maruca vitrata* Fabricius, Lepidoptera: Pyralidae) [23] and pea aphids (*Acyrthosiphon pisum* Harris, Aphididae: Macrosiphini) in plants [24]. In brief, AMF may be closely associated with the concentration of flavonoids and phenolics in plants, thus influencing plant defense against pest insects. Additionally, inoculation with AMF increases plant growth [25] and nutrient content, which help compensate for the damage caused by pests [26]. Whereas, the symbiotic relationship between AMF and the host is controlled by soil nutrition, which suggests that optimal fertilization should be taken into consideration to simultaneously improve plant growth, crop yield, and pest management through the benefits of AMF.

Nitrogen is an essential macronutrient that promotes plant development and crop yield and quality [27,28,29,30]. Many studies have claimed that N is an essential element involved in the biosynthesis of chlorophyll [31], nucleic acids [32], amino acids [33], and enzymes [34], which are associated with the accumulation of plant nutrients [35,36]. In addition, N is the key factor for the metabolism of plant secondary chemicals, such as phytohormones (e.g., abscisic acid, ABA; indole-3-acetic acid, IAA; cytokinin, CK) [37,38], polyphenolics, tannins, and flavonoids [39,40,41,42]. For example, total phenolics and total flavonoids increased with an increase in nitrogen fertilizer application [43,44]. However, excessive amounts of N fertilizer in the soil (e.g., 350 kg N/ha) may result in decrease AMF colonization of plant roots, because nitrogen addition may suppress the sporulation of AMF in some gramineous species [45], and decrease the abundance of AMF [46]. High amounts of N fertilizer may also increase the abundance of aphids in wheat [8,41]. For instance, the population size, fecundity and longevity of several aphids (e.g., *Rhopalosiphum padi* L. and *Sitobion avenae* F.) were greater at higher amounts of N fertilizer [47,48]. Especially for the *S. avenae,* Aqueel (2011), which suggests that N-fertilizer had a positive effect on the weight, fecundity and longevity [48]. Therefore, appropriate N management needs to be tested to benefit wheat growth.

Phosphorus (P) is another important nutrient, required in relatively large amounts for the biosynthesis of primary and secondary metabolites [49], because it is an essential element for the formation of nucleic acids and phospholipids. It also plays a key role in the energy metabolism of photosynthetic processes [50]. A study on wheat found that P fertilizer reduced the concentration of phenolic acid and flavonoids in leaves [51]. The reason may be due to a reduction of AMF colonization in the soil in the presence of relatively high amounts of P. Many reports demonstrate that the content of P in soil is negatively correlated with AMF [16,17] and affects the secondary metabolism of the host. Hence, a better understanding of how P fertilization interacts with AMF is important for developing an optimum fertilization model.

**Figure 1:**
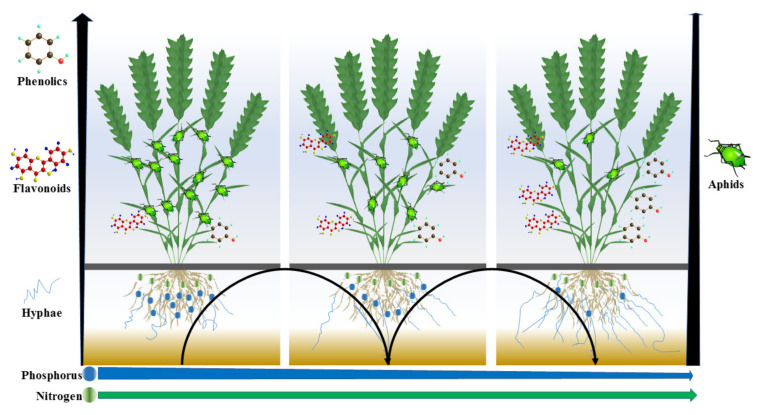
The hypothesis of this study, which claimed that decreasing the phosphorus amount will result in arbuscular mycorrhizal fungi (AMF) colonization of wheat roots. An increase in AMF colonization might play some important roles in nutrient absorption, the metabolism involved in insect resistance, and even a reduction of nitrogen and phosphorus residues in soil.

Considering the important role of AMF in regulating soil nutrient uptake, plant secondary metabolites, and pest insect populations, knowledge of the optimal amount of N and P is key to plant protection by improving AMF colonization (Figure 1). Here, we conducted both garden and field experiments to (1) determine the effects of different ratios of P to N on AMF colonization on wheat roots, (2) evaluate the effect of the preferred fertilizer regime on wheat aphids (*S. avenae*), and (3) identify the secondary chemicals that may be associated with wheat resistance to aphids.

## 2. Materials and Methods

### 2.1. Study Sites

The studies were conducted at Henan University, Kaifeng, Henan Province, China (E: 114.23, N: 34.52, altitude 73 m). Kaifeng is a city with a temperate monsoon climate. In winter and spring, the average temperature was 2–11 °C and 6–15 °C (http://www.tianqi.com/qiwen/city-kaifeng), with dry air and wind, respectively. The annual precipitation was 670 mm, and the rainy season was from March to October.

### 2.2. Soil Preparation

To ensure the same nutrient conditions in the experiment plots, we collected the top 50 cm of soil by using excavator (HYUNDAI Industries Co., Ltd., Taian, Shandong, China), and then mixed these soils 6 times using a wheel loader (Caterpillar Ltd., Qingzhou, Shandong, China), before they were used to be carried out field and common garden experiment. The dry homogenized soil contained 1.41 g of total nitrogen, 0.48 g of nitrate nitrogen, 0.51 g of ammonia nitrogen, 0.06 g of total carbon, and 0.43 g of phosphorus per kg of soil. The pH value was neutral (around 7.0–7.3).

### 2.3. Study Materials

The cultivar of winter wheat (*Triticum aestivum* L.) used in this study (both the common garden and field experiment) was Zhoumai 22, which is a hybrid of Zhoumai 12, Wenmai 6, and Zhoumai 13, and developed by the Zhoukou Academy of Agricultural Sciences, Zhoukou city, Henan Province, China. This cultivar is widely grown on the central plains of China, such as the Yellow and Huaihe River basins, because of its strong cold tolerance and disease resistance [52].

The aphid specie, *Sitobion avenae*, was selected to be used in this study. *S. avenae* is the dominant species in wheat plants in the North of China [53], Europe, and America [54], it usually feeds on the wheat plants from jointing stage to maturation stage in Kaifeng city, and it causes damage to wheat plants, not only by feeding, but also by spreading lots of pathogen. In this study, the tested aphids were collected from a wheat field in Rice Village (E: 114.34 N: 34.88), where Zhoumai 22 is mostly planted. They were fed with the seedlings of the same wheat species at 20 °C, 75% RH (relative humidity), and 16:8 light/dark conditions for 7 days. We selected urea and monobasic potassium phosphate as the sources of N and P, respectively. These fertilizers were bought from the Sinochem Fertilizer Co., Ltd., Beijing, China, and the content of urea and monobasic potassium phosphate exceeded 99%.

### 2.4. Experimental Design

To test the effect of soil P and N on AMF colonization, aphid abundance, and secondary chemicals in wheat plants, we carried out a common garden experiment from October 2017 to May 2018 in an open-sided greenhouse. In the common garden experiment, 90 flowerpots (diameter 25 cm and height 28 cm) were prepared to be filled with 8.5 kg of the prepared soil, and then we sowed 10 seeds into each pot. After germination, we selected five similar-sized seedlings as test plants, by removing the other seedlings when their fourth leaf appeared. We then applied 1000 mL of different combinations of P and N fertilization (for details, see Table 1) into these pots each month to maintain the tested level of N and P. Ten pots per combination of P and N fertilization as 10 repeats. In total, 9 combinations (treatments) in the study, which included P0 and N0 (P0N0), P0 and N1 (P0M1), P0 and N2 (P0H2), P1 and N0 (P1N0), P1 and N1 (P1N1), P1 and N2 (P1H2), P2 and N0 (P2N0), P2 and N1 (P2N1), and P2 and N2 (P2N2). In addition, we inoculated five aphids (*S. avenae*) onto seedlings in each pot on the 1 May 2018, and used nylon mesh (16 openings/cm) to prevent the tested aphids from escaping, and to protect the plants from other insects in the meantime. At the end of May, before these tested plants began to yellow, we counted the number of aphids on all tested plants in each pot, and then collected the green leaves and stem to measure the total phenolics and total flavonoids in the tested plant leaves, as well as the aboveground biomass after drying at 45 °C in an oven (Shanghai Boxun Industry & Commerce Co., Ltd., medical equipment factory, Shanghai, China). We washed the roots from the pots in tap water to test the AMF colonization rate, as described below. Before this study, we also prepared 45 flowerpots (9 soil nutrition treatments and 6 repeats per treatment, 1 pot as a repeat), which were used to carry out the research on the effect of different soil nutrition (same treatments to that with *S. avenae*) on plant performance without any stress. The observed results included AMF colonization and total flavonoids and total phenolics in leaves (unpublished before).

To test the effect of P and N on wheat growth in the field (without any insects), we conducted a controlled experiment in farmland (30 × 30 m) near the common garden experiment at the same time. The field trial included 54 test plots (9 treatments and 6 repeats per treatment), which was 4.5 m × 2.5 m per plot, and these plots were spaced 50 cm apart and were divided by iron sheets driven into the ground to a depth of 50 cm. Before irrigating these plots by tap water and sowing seeds, these plots were filled with the prepared soil, and randomly selected to be treated with 9 combinations of N and P (see details in Table 1). These combinations (treatments) also included P0 and N0 (P0N0), P0 and N1 (P0M1), P0 and N2 (P0H2), P1 and N0 (P1N0), P1 and N1 (P1N1), P1 and N2 (P1H2), P2 and N0 (P2N0), P2 and N1 (P2N1), and P2 and N2 (P2N2). The chemical fertilizers were applied by hand based on weight per plots. Six replications per treatment were prepared for each combination. We sowed the wheat seeds by hand, with seven rows per plot (150 g/plot). Plants were protected from herbivory with nylon mesh (16 openings/cm) cages that fitted over each plot. AMF colonization rates of wheat roots, aboveground biomass (dry weight of leaves and stems), carbon and nitrogen contents in dry leaves were determined in 15 plants, which were selected randomly before harvesting, from each plot. The wheat yield in each plot were evaluated by the dry weight of seeds from one square meter.

### 2.5. Arbuscular Mycorrhizal Fungi (AMF) Colonization Rate

The AMF colonization rate of wheat roots was determined following the methods described by Giovannetti [55]. The procedure was carried out as follows: the selected roots were cleared in 2.5% KOH at 80 °C, before acidizing with 2% HCl. The fungal structures were stained with 0.05% Trypan blue for 24 h, after being washed in tap water five times. We then washed the Trypan blue out of the test roots and counted the hyphae at each of 300 gridline intersections on thirty 1 cm fine root segments, all from a single flowerpot, at 200 × microscope magnification [56,57,58].

### 2.6. Chemical Analysis

We analyzed the concentration of total phenolics and total flavonoids in wheat leaves with a spectrophotometer (Thermo GENESYS 10S, Waltham, MA, USA). The tested leaves were dried at 40 °C for 72 h and then ground with a ball mill (Heng’ao HMM-400A, Tianjin Heng’ao Technology Development Co., Ltd. Tianjin, China), before chemical analysis. The total phenolics and flavonoids in 100 mg of leaf powder were extracted for 24 h in 3 mL of methanol −0.4% phosphoric acid in water solution (48:52, *v*/*v*). We collected these solutions after centrifugation at 5000 rpm.

The concentration of total phenolics was measured as follows. The reaction mixture was prepared by mixing 0.5 mL of sample, 2.5 mL of 10% Folin–Ciocalteu reagent, and 2 mL of 7.5% NaHCO_3_. The blank was a mixture of 0.5 mL of methanol, 2.5 mL of 10% Folin–Ciocalteu reagent, and 2 mL of 7.5% of NaHCO_3_. These samples were tested using a spectrophotometer at 765 nm after being incubated at 45°C for 45 min. The standard curve was phenol [59]. The concentration of flavonoids in the leaves was determined as follows. We mixed 1 mL of sample solution and 1 mL of 2% AlCl_3_ solution dissolved in methanol, and then let it incubate for 60 min at room temperature. We used the spectrophotometer to test the absorbance of the reaction mixture at 275 nm. The content of flavonoids in extracts was expressed in terms of rutin equivalent [60]. The concentration of carbon and nitrogen in leaves was determined with a Vario MACRO cube element analyzer (Elementar, Hanau, Germany) after these dry samples were wrapped in silver paper [61].

### 2.7. Data Analysis

Two-way analysis of variance (ANOVA) was used to analyze the main and interactive effects of P and N on all the collected data in the common garden experiment and the field trial. For aphid abundance, we calculated the mean number of aphids on the stems from the same plants. Then, one-way ANOVA was performed to analyze the effect of P or N on the total flavonoids and aboveground biomass at each level of N or P, because there was an interaction effect on them between N and P. The Tukey test was used to determine the significant differences at *p* < 0.05. These statistical analyses were conducted with SAS 9.4 software (SAS Institute Inc., Cary, NC, USA).

In order to explore whether there was a relationship among the soil nutrition, AMF, and pest resistance, we performed structural equation modeling (SEM), based on the hypothesis that less P application enhances wheat resistance to aphids by increasing AMF and some secondary chemicals using the Amos package with SPSS 22.0 (Amos Development Corporation, Meadville, PA, USA) software. The data (aphid abundance, AMF colonization rate, total flavonoids, and total phenolics) we collected in the garden experiments were from the same plants, and the aphid abundance was the mean number of aphids on the stems from the same plants. The goodness of fit of the method was evaluated based on the following indices: chi-square goodness-of-fit statistic (χ^2^), goodness fit index (GFI), and the root mean squared error of approximation (RMSEA) [62].

## 3. Results

### 3.1. Effect of P and N Fertilizer Amounts on AMF

**Figure 2:**
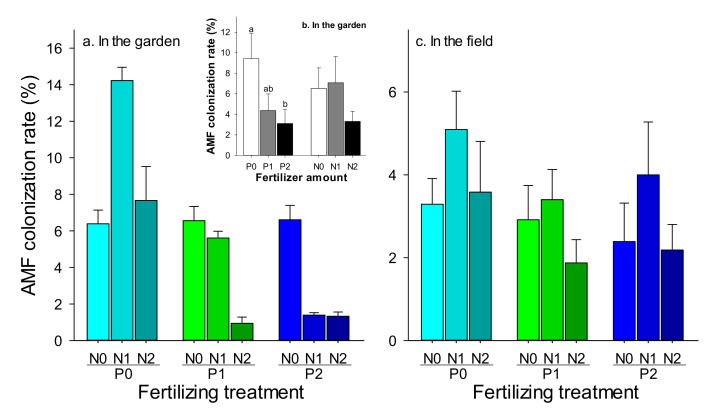
The effect of different combinations (amounts) of phosphorus (P) and nitrogen (N) fertilization on the AMF colonization rate of wheat roots in the garden and the field. (**a**) The effect of combinations of P and N Fertilization, (**b**) the effect of different amount of P or N based on the two-way analysis of variance (ANOVA) that indicated there was no interaction between P and N, (**c**) the effect of combinations of P and N Fertilization in field. The bars are the means with SE. Bars with different letters indicate significant differences based on one-way ANOVA at *p* < 0.05.

With respect to the effect of fertilization on the AMF colonization rate, there was no interaction between N and P in both the garden and the field (Table 2). The higher AMF colonization rate was measured in the plants that were treated with P0 and N1 in both the garden (14.22%) (Figure 2a) and the field (5.09%) (Figure 2c), and these values were higher than those of other treatments by at least 20% (Figure 2a,c). Further simple effect analysis showed that the AMF colonization rates on wheat roots increased significantly in the treatment without P fertilization (P0), compared to that with P1 and P2 in the garden (Figure 2b), but not found in the field (Table 2).

### 3.2. Effect of P and N Fertilizer Amounts on Wheat Aphids

**Figure 3:**
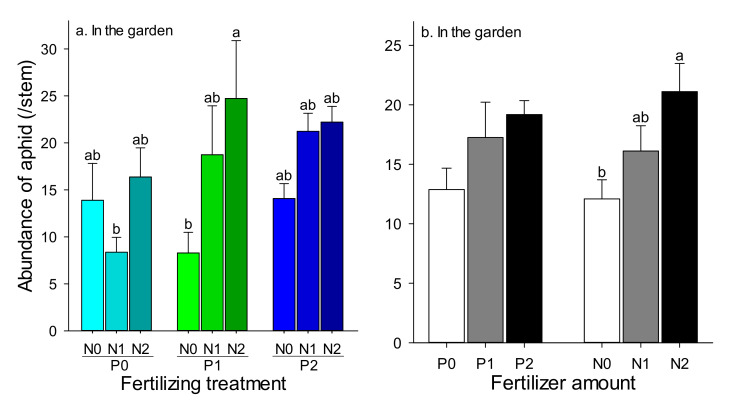
The effect of different amounts of phosphorus (P) and nitrogen (N) fertilization on aphid abundance in the garden. (**a**) the effect of combinations of P and N Fertilization, (**b**) the effect of different amount of P or N based on the two-way ANOVA. which indicated there was no interaction between P and N. The bars are the means with SE. Bars with different letters indicate significant differences based on one-way ANOVA at *p* < 0.05.

In the common garden experiment, there was no interaction between P and N fertilizer with respect to the abundance of *S. avenae* (Table 3). The lower abundances of aphid were happened in the P0N1 (8.37 aphid) and P1N0 (8.29 aphid) (Figure 3a). The level of N fertilizer was the key factor that determined aphid abundance (Table 3). Aphid abundance was increased with the increase in N fertilizing, and the aphid abundance was higher in the treatment with N2, than that with N0 significantly (Figure 3b). There was no significant difference among these treatments with different levels of P fertilization, although the aphid abundance also increased with an increase in the fertilizer amount (Figure 3b).

### 3.3. Effect of P and N Fertilizer Amounts on Secondary Metabolites in Wheat Leaves

**Figure 4:**
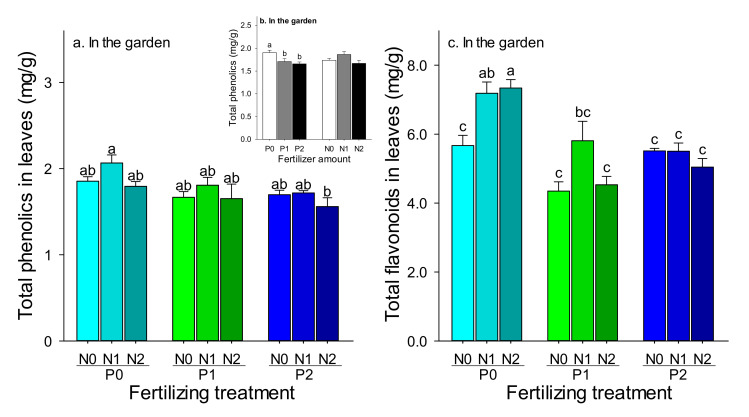
The effect of different amounts of phosphorus and nitrogen fertilization on total phenolics (**a**,**b**) and total flavonoids (**c**). (**a**,**c**) the effect of combinations of P and N Fertilization, (**b**) the effect of different amount of P or N based on the two-way ANOVA. The bars are the means with SE. Bars with different letters indicate significant differences based on one-way ANOVA at *p* < 0.05.

In the garden experiment, with respect to the effect on total phenolics, there was no interaction between P and N fertilizer (Table 4). The higher concentration of total phenolics was found in P0N1 (2.07 mg/g) (Figure 4a). There was a significantly higher concentration of total phenolics in P0 compared to P2 (Figure 4b), but no significant difference in the pots treated with N (Figure 4b). With respect to the effect on total flavonoids, there was a significant interaction between P and N (Table 4). The results based on simple effect analysis showed that a higher concentration of total flavonoids was found in P0N1 (7.18 mg/g) and P0N2 (7.33 mg/g), compared with the other treatments, which were higher by at least 23.7%) (Figure 4c). In addition, there were similar results in the control (Table S1), which indicated the level of P had significant effect on the phenolics and (Table S1 and Figure S1a), and the P and N had significant effect on the flavonoids (Table S1 and Figure S1b).

### 3.4. Effect of P and N Fertilizer Amounts on Plant Traits

There was a significant interaction between N and P fertilizer in both the garden and the field for aboveground biomass (Table S2). In the garden, higher aboveground biomass of wheat was observed in treatments P0N1 (0.56 g DW) and P2N1 (0.57 g DW), and these values were higher than those of other treatments by at least 10.6% (Figure S2a). In the field, higher aboveground biomass was observed in treatments P1N2 (2.6228 g DW), P2N2 (2.3118 g DW), and P0N1 (2.2928 g DW), these values were higher than those of other treatments by at least 24.3% (Figure S2b). However, there was no significant interaction between N and P in the field, with respect to wheat yield and the C/N ratio in leaves (Table S3). Furthermore, only yield increased with increasing amounts of N, and was significantly lower in the N0 treatment than in the N1 and N2 treatments (Figure S3a). With respect to the C/N ratio in leaves, there was no significant difference among these treatments (Figure S3b).

### 3.5. Structural Equation Modeling (SEM)

**Figure 5:**
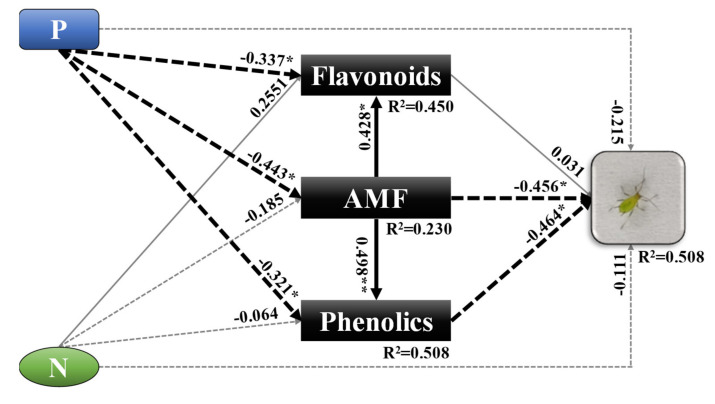
Structural equation modeling (SEM) indicated the direct and indirect effects of the amounts of nitrogen and phosphorus fertilization on AMF, aphids, flavonoids, and phenolics in wheat leaves. Numbers adjacent to arrows are the pathway coefficients and the effect size of the relationship. Continuous arrows mean positive relationships, dashed arrows mean negative relationships, thin and gray lines mean no significant relationship at the 5% level (*p* > 0.05), and thick and black lines mean a significant relationship at the 5% level (*p* < 0.05). Significance is indicated by * *p* < 0.05, ** *p* < 0.01. The model created was satisfactorily fitted to our data based on the following values: χ^2^ = 0.849; GFI = 1.000; RMSEA = 0.000; *p* = 0.357. In the pathway, N is nitrogen, P is phosphorus, AMF represents arbuscular mycorrhizal fungi, aphid represents the number of *S. avenae*, and flavonoids and phenolics represent the respective concentrations (mg/g) of total flavonoids and total phenolics in dry leaves.

In the SEM, we investigated the direct and indirect relationships among P and N fertilizer amounts, AMF, metabolism, and aphid abundance. The indirect, direct, and total pathway effects of P and N on AMF and aphids were studied in detail. In brief, a lower level of P fertilizer directly enhanced AMF colonization on wheat roots, and then negatively affected aphid abundance. Furthermore, the increase in AMF increased the concentration of total flavonoids and total phenolics in leaves, and then suppressed the aphid abundance by phenolics, due to the negative relationship between phenolics and aphids (Figure 5).

## 4. Discussion

In this study, we examined the effects of N and P on AMF colonization rates, wheat growth and yield, and aphid population density. We found different combinations of N and P resulted in different AMF colonization rates. Generally, the P0N1 treatment significantly increased AMF colonization and secondary chemicals (flavonoids and phenolics), but reduced the numbers of wheat aphids and had no effect on wheat growth and yield. Therefore, this treatment may be considered optimum.

The response of wheat growth to soil nutrient levels has received much attention. Many studies have shown that N is a major limiting factor in crop production, with a positive relationship between wheat yield and N amount [63,64,65]. Our results are consistent with these studies, and we found that aboveground biomass and yield increased with increasing N. Such effects of N on wheat yield are reasonable, because increasing N levels significantly increase the grain count, the number of spikes, and the thousand-grain weight [66]. At same time, many studies have found that AMF colonies play an important role in plant nutrient absorption, including soil N and P [67]. Therefore, enhancing the interaction between AMF and wheat is an important way to promote wheat growth and yield [68,69,70], without increasing the amount of fertilizer applied.

Previous studies on the effects of soil nutrients on insects reported that N enhances the population density of aphids because nitrogen in plants induces more amino acids [71], which positively affect aphids [72], and our results in this study are in line with these previous findings. Wilkinson et al. (2019) reported that AMF can deliver N from organic sources to the plant, but has no effect on aphid number [73]. The reason may be that there are more secondary metabolites in plants with high AMF colonization [74], such as flavonoids and phenolics, which could provide strong resistance against aphids [75,76,77]. In addition, changes in soil N may affect the content of flavonoids [78,79] and phenolics [80,81]. Some studies have also shown that reducing the amount of P fertilizer results in an increase in the content of total flavonoids [82,83,84], and phenols were positively correlated with maximal photosynthesis at lower amounts of P [85]. Additionally, reducing P results in a high AMF colonization rate of plant roots [86], which can improve photosynthesis [87], these reports are also proved by our results, especially in the common garden experiments. There was no significant effect of P on the AMF colonization in field trial, which may be due to the heavy rain in this year, because previous report suggests that the mean colonization rate in wet soils is lower than dry soils by 35.29% [88]. Regardless, these findings are line with the SEM results, which showed a negative relationship between the amount of P and AMF and a positive relationship between AMF and the concentrations of total phenolics and flavonoids.

Furthermore, the SEM showed a negative relationship between the concentration of total phenolics and aphid abundance, as well as between AMF and aphid abundance, but a positive relationship between AMF and total phenolics, which means that AMF may enhance the resistance of wheat to aphids through secondary metabolites [89,90]. Together with the similar results of the secondary chemicals were observed in control, which indicated the secondary chemicals were not affected by aphid (Table S3 and Figure S3), we can conclude that higher AMF colonization of wheat roots, nutrition absorption, and pest resistance occurred, which made the wheat plants grow well even at lower levels of P fertilization [91]. These findings provide critical evidence for how the amount of soil N and P can impact wheat aphids directly and indirectly.

In brief, our findings provided new insights into how AMF, aphids, and wheat growth respond to varying levels of N and P in soil, although the complex mechanism was not very clear. Over the past few years, field and laboratory studies have shown that wheat yield and aphids respond strongly to soil fertilization [92]. Furthermore, it has been shown that wheat yield and aphids are also sensitive to AMF abundance [93,94]. For wheat, it is well known how yield and aphids are affected by N or P alone, but the response of the wheat growth and wheat aphids to the combination of N and P remains largely unknown, and this needs to be addressed in a future study.

## 5. Conclusions

In this study, we found that AMF abundance, wheat growth, wheat aphids, and secondary metabolites showed different responses to the varying combinations of N and P fertilizer, which were distinct from the plant response to N or P alone in previous studies. Further field and laboratory studies are needed to understand the mechanisms of the effect of N and P combinations and to make clear the direct and indirect interactions among plant nutrients, AMF levels, and aphid abundance. We recommend that future studies on the effects of soil nutrition on crop growth, yield, and aphids consider the responses of the complex combination of soil nutrition in different soils. This approach will also help decrease the need for chemical fertilizer and prevent pollution of the agroecological environment.

## Figures and Tables

**Table 1:** The amounts of nitrogen (N) and phosphorus (P) fertilization based on the active ingredient of chemical fertilizers (P = monobasic potassium phosphate, N = urea).

Nutrient Source	Levels	Fertilizer Amount	
		In the Garden (g/pot)	In the Field (g/plot)
Monobasic potassium Phosphate (P)	P0	0	0
	P1	0.8172	187.67
	P2	1.6344	375.34
Urea (N)	N0	0	0
	N1	1.3083	299.84
	N2	2.6166	599.68

**Table 2:** The dependence of the arbuscular mycorrhizal fungi (AMF) colonization rate on the amounts of phosphorus (P0, P1, P2) and nitrogen (N0, N1, N2) fertilization and their interaction in the garden and the field. Significant results are indicated in bold.

Effects	df	In the Garden		In the Field	
		F	P	F	P
Phosphorus (P)	2	**3.21**	**0.0499**	1.96	0.1492
Nitrogen (N)	2	3.29	0.3160	2.94	0.0605
P*N	4	1.13	0.3564	0.23	0.9187

**Table 3:** The dependence of aphids on the amounts of phosphorus (P0, P1, P2) and nitrogen (N0, N1, N2) fertilization and their interaction in the garden. Significant results are indicated in bold.

Effects	df	F	P
Phosphorus (P)	2	2.65	0.0770
Nitrogen (N)	2	**5.18**	**0.0076**
P *N	4	1.88	0.1211

**Table 4:** The dependence of total flavonoids and total phenolics on the amounts of phosphorus (P0, P1, P2) and nitrogen (N0, N1, N2) fertilization and their interaction in the garden. Significant results are indicated in bold.

Effects	df	Total Phenolics		Total Flavonoids	
		F	P	F	P
Phosphorus (P)	2	**6.58**	**0.0072**	**29.63**	**<0.0001**
Nitrogen (N)	2	3.78	0.0425	**7.95**	**0.0034**
P*N	4	0.38	0.8216	**4.45**	**0.0113**

## Supplementary Materials

The following are available online at https://www.mdpi.com/2075-4450/11/6/365/s1, Table S1: The dependence of the phenolics and flavonoids on the amounts of phosphorus (P0, P1, P2) and nitrogen (N0, N1, N2) fertilization and their interaction in control in the garden. Significant results are indicated in bold, Table S2: The dependence of aboveground biomass on the amounts of phosphorus (P0, P1, P2) and nitrogen (N0, N1, N2) fertilization and their interaction in the garden and the field. Significant results are indicated in bold, Table S3: The dependence of wheat yield (per m2) and the C/N ratio on the amounts of phosphorus (P0, P1, P2) and nitrogen (N0, N1, N2) fertilization and their interaction in the field. Significant results are indicated in bold, Figure S1: The effect of different combination of phosphorus and nitrogen fertilization on the total phenolics (a) and total flavonoids (b) in leaves in the control. The bars are the means with SE. Bars with different letters indicate significant differences at *p* < 0.05, Figure S2: The effect of different combination of phosphorus and nitrogen fertilization on the aboveground biomass of wheat plant in both the garden (a) and the field (b). The bars are the means with SE. Bars with different letters indicate significant differences at *p* < 0.05, Figure S3: The effect of different combination of phosphorus and nitrogen fertilization on wheat yield (a) and the C/N ratio (b) in the field. The bars are the means with SE. Bars with different letters indicate significant differences at *p* < 0.05.
