# Supplementary material for: Effect of Different Combinations of Phosphorus and Nitrogen Fertilization on Arbuscular Mycorrhizal Fungi and Aphids in Wheat
Source: Insects. 2020 Jun 11;11(6):365. doi: 10.3390/insects11060365 (PMC7349843; doi:10.3390/insects11060365)
Supplement: Supplementary file 1 [file insects-11-00365-s001.pdf]

Table S1. The dependence of the phenolics and flavonoids on the amounts of phosphorus (P0, P1, P2) and nitrogen (N0, N1, N2) fertilization and their interaction in the control. Significant results are indicated in bold.

Effects	df	Total Phenolics		Total Flavonoids	
		F	P	F	P
Phosphorus (P)	2	**18.02**	**<0.0001**	**10.00**	**0.0012**
Nitrogen (N)	2	**3.70**	**0.0451**	**5.94**	**0.0104**
P *N	4	1.29	0.3109	**3.62**	**0.0248**

Table S2. The dependence of aboveground biomass on the amounts of phosphorus (P0, P1, P2) and nitrogen (N0, N1, N2) fertilization and their interaction in the garden and the field. Significant results are indicated in bold.

Effects	df	In the Garden		In the Field	
		F	P	F	P
**Phosphorus (P)**	2	**3.63**	**0.0403**	0.89	0.4128
Nitrogen (N)	2	3.29	0.0528	**6.51**	**0.0022**
P *N	4	**5.63**	**0.0020**	**3.20**	**0.0163**

Table S3. The dependence of wheat yield (per m^2) and the C/N ratio on the amounts of phosphorus (P0, P1, P2) and nitrogen (N0, N1, N2) fertilization and their interaction in the field. Significant results are indicated in bold.

Effects	df	Yield		C/N Ratio	
		F	P	F	P
Phosphorus (P)	2	0.05	0.9504	2.19	0.1220
Nitrogen (N)	2	**26.67**	**<0.0001**	0.94	0.3982
P *N	4	2.00	0.0997	0.40	0.8055

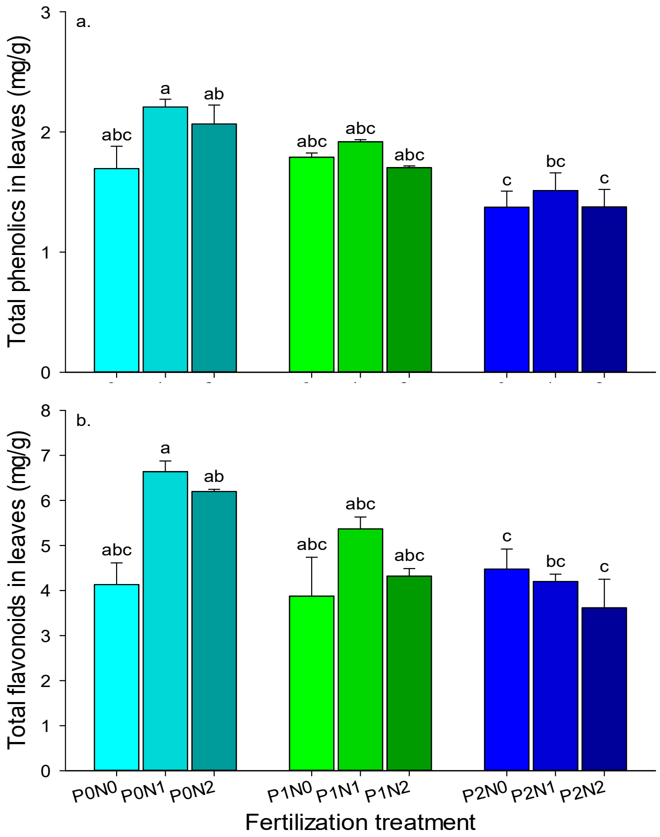

Figure S1. The effect of different combinations of phosphorus and nitrogen fertilization on the total phenolics (**a**) and total flavonoids (**b**) in leaves in the control. The bars are the means with SE. Bars with different letters indicate significant differences at $p < 0.05$.

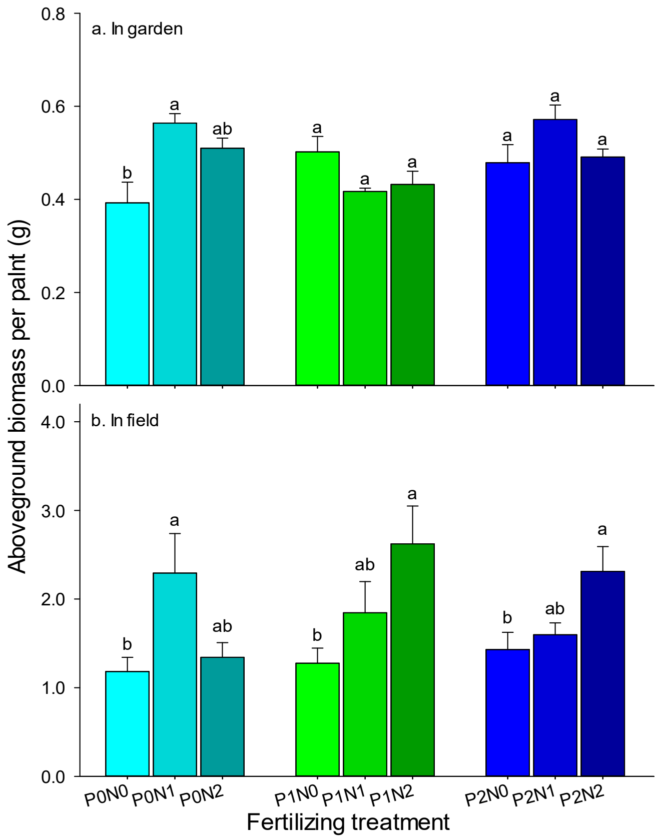

Figure S2. The effect of different combinations of phosphorus and nitrogen fertilization on the aboveground biomass of wheat plant in both the garden (**a**) and the field (**b**). The bars are the means with SE. Bars with different letters indicate significant differences at $p < 0.05$.

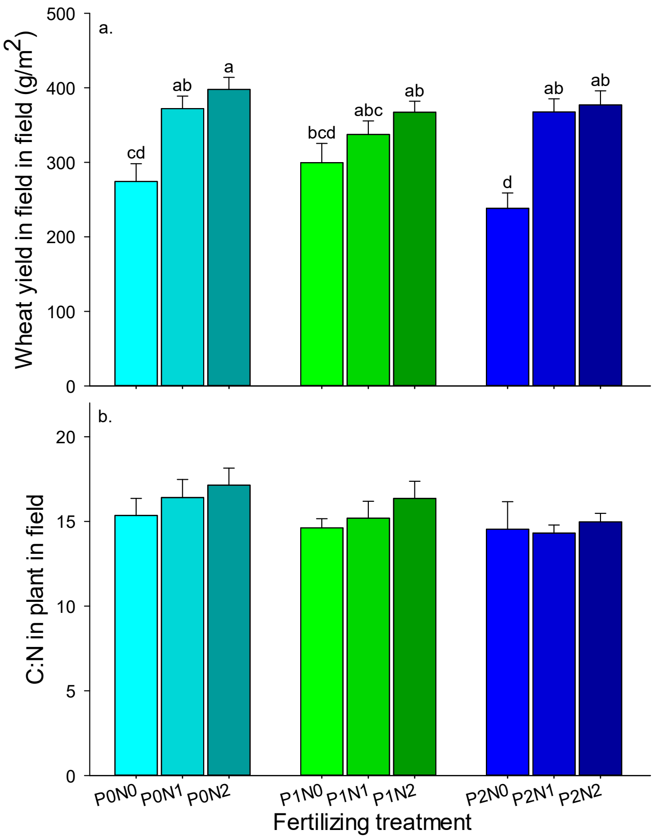

Figure S3. The effect of different combinations of phosphorus and nitrogen fertilization on wheat yield (**a**) and the C/N ratio (**b**) in the field. The bars are the means with SE. Bars with different letters indicate significant differences at $p < 0.05$.
